# 5-{4-[(1*R*,3*S*,4*S*)-Neomenth­yloxy]phen­yl}-1*H*-tetra­zole

**DOI:** 10.1107/S2414314626000337

**Published:** 2026-01-16

**Authors:** Heiner Detert, Dieter Schollmeyer

**Affiliations:** a University Mainz, Duesbergweg 10-14, 55099 Mainz, Germany; Goethe-Universität Frankfurt, Germany

**Keywords:** crystal structure, heterocycle, hydrogen bonds

## Abstract

The monoclinic unit cell of the title compound, C_17_H_24_N_4_O, contains four mol­ecules, two *A* and two *B*. In the extended structure, the two similar conformers are connected into *A*-ribbons and *B*-ribbons. Three hydrogen bonds connect the mol­ecules within the ribbons, while π–π inter­actions between the phenyl and tetra­zole moieties of different mol­ecules [centroid–centroid distances of 3.5288 (11) and 3.6138 (11) Å] connect the *A* and *B* strands; the aromatic rings are nearly coplanar.

## Abstract 2

The monoclinic unit cell of the title compound contains four mol­ecules, two *A* and two *B*. In the extended structure, the two similar conformers are connected into *A*-ribbons and *B*-ribbons. Three hydrogen bonds connect the mol­ecules within the ribbons, while π–π inter­actions between the phenyl and tetra­zole moieties of different mol­ecules connect the *A* and *B* strands; the aromatic rings are nearly coplanar.

## Graphical abstract

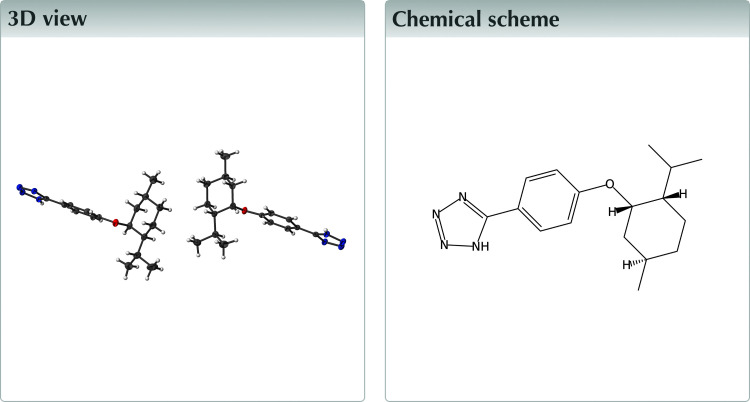

## Structure description

**Figure 1:**
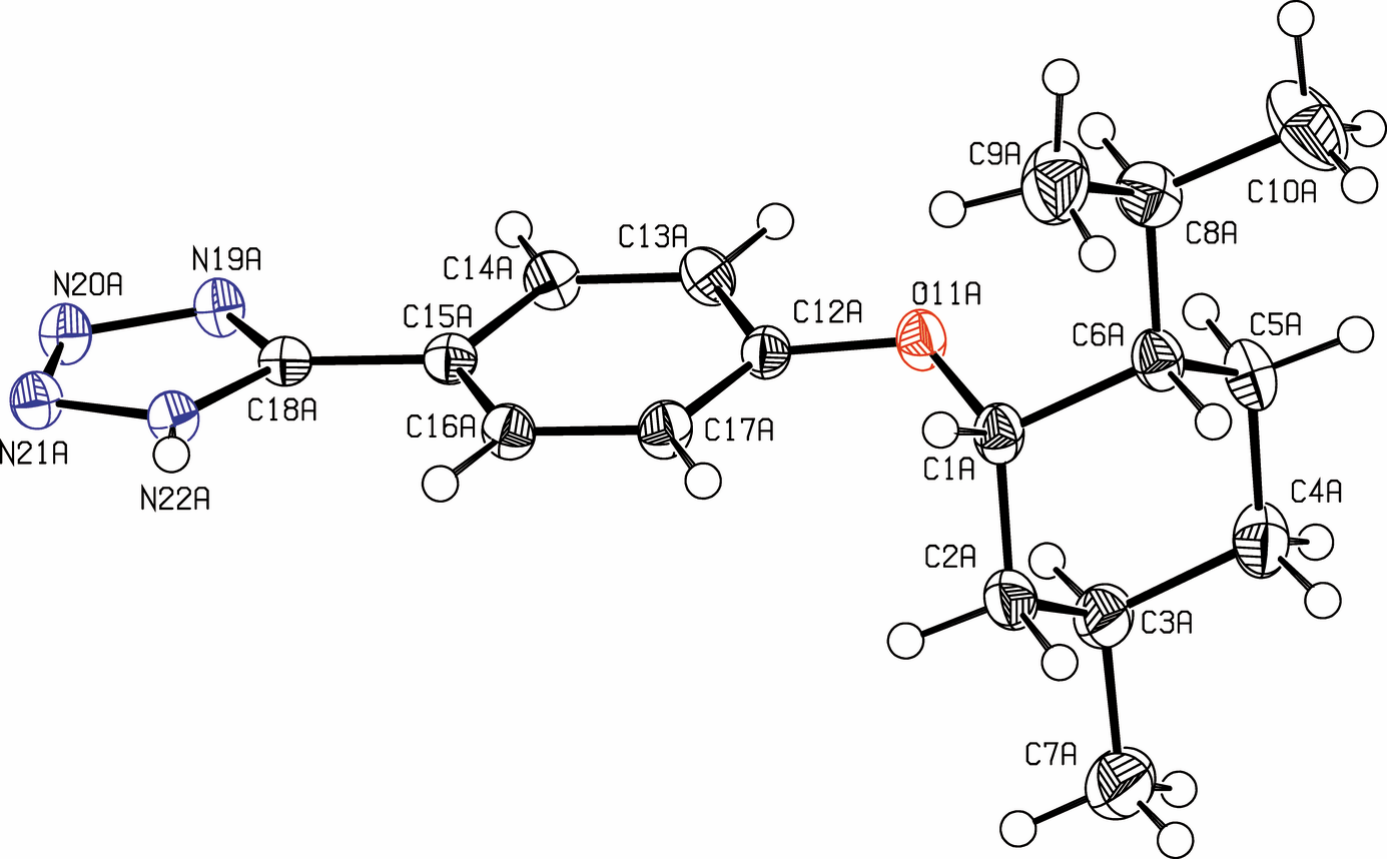
View of the title compound. Displacement ellipsoids are drawn at the 50% probability level. Only one of the two independent mol­ecules is shown.

The title compound (Fig. 1 ▸) was prepared in a larger project on discotic liquid crystals (Rieth *et al.*, 2018 ▸; Tober *et al.*, 2019 ▸; Graschtat *et al.*, 2025 ▸). The condensation of tetra­zoles with cyanuric chloride leading to tris­triazolotriazines was discovered by Huisgen (Huisgen *et al.*, 1961 ▸) and successfully applied for the synthesis of liquid crystals (Cristiano *et al.*, 2008 ▸; Rieth *et al.*, 2020 ▸). The title compound, C_17_H_24_N_4_O, was used as starting material for the synthesis of tris-(neomenthyloxyphen­yl)-tris­triazolotriazine (Herget *et al.*, 2013 ▸). The monoclinic unit cell contains four mol­ecules, two *A* and two *B. A* and *B* differ only slightly in their conformation, the main difference being the torsion angle at the neomenth­oxy ether linkage. This angle (C6—C1—O11—C12) in *A* is −164.63 (15)° whereas in *B* the torsion angle amounts to −142.09 (16)°. In both mol­ecules, the phenyl and tetra­zole rings are nearly coplanar [dihedral angle: 6.99 (11)° in mol­ecule *A* and 1.53 (11)° in mol­ecule *B*] and the aryl­ether group is only weakly bent [C13*A*—C12*A*—O11*A*—C1*A* = −169.86 (16)° and C13*B*—C12*B*—O11*B*—C1*B* = 168.60 (16)°]. The cyclo­hexane adopts a chair conformation with equatorial alkyl groups and an axial ether linkage. Mol­ecules *A* and *B* are anti­parallel and they are connected *via* π–π inter­actions between the phenyl and tetra­zole moieties. These rings are almost parallel, dihedral angles between the least-squares planes are tetra­zole *A*/phenyl *B =* 0.97 (10)° and tetra­zole *B*/phenyl *A* = 8.05 (10)°. The distance between the centroids of phenyl *A* and tetra­zole *B* rings is 3.5288 (11) Å and that between phenyl *B* and tetra­zole *A* is 3.6138 (11) Å.

**Figure 2:**
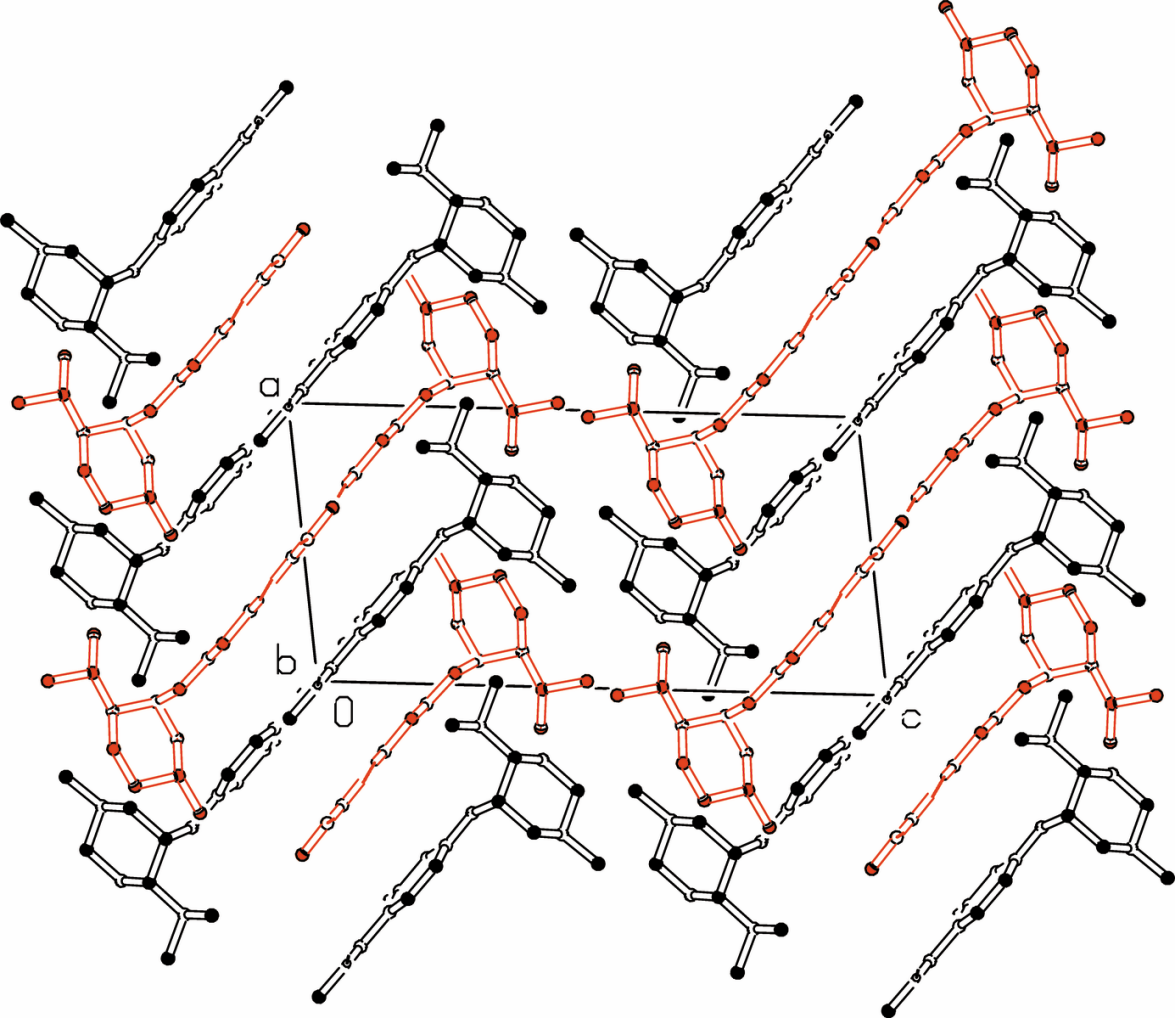
Partial packing diagram. View along the *b*-axis direction. Only hydrogen atoms involved in hydrogen bonds are shown.

In the extended structure (Fig. 2 ▸), three hydrogen bonds connect *A* mol­ecules into ribbons and, in a very similar manner, the *B* mol­ecules into ribbons. Both ribbons run along the *b*-axis direction and they are nearly *C*2-symmetrical. In the strands, the mol­ecules are geometrically connected *via* the 2_1_ axis. The inter­molecular hydrogen bonds are N22—H22⋯N19 with distances of N22*A*—H22*A =* 0.82 (3) Å, H22*A*⋯N19*A =* 2.08 (3) Å and N22*A*⋯N19*A =* 2.903 (3) Å, the bond is close to be linear: 177 (2)°. Two C—H⋯N bonds (C14—H14⋯N21, C16—H16⋯N21) are significantly longer and bent: C14*A*—H14*A =* 0.95 Å, H14*A*⋯N21*A =* 2.57 Å, C14*A*⋯N21*A =* 3.495 (3) Å, angle C—H⋯N = 165.2° and C16*A*—H16*A =* 0.95 Å, H16A⋯N20*A =* 2.51 Å, C16*A*⋯N20*A =* 3.423 (3) Å, angle C—H⋯N = 160.4° (for values for mol­ecule *B*, see Table 1 ▸).

## Synthesis and crystallization

The two-step synthesis of the title compound was performed *via* Mitsunobu reaction of *p*-cyano­phenol (0.1 mol) and (−)-menthol (0.12 mol) using diethyl azodi­carboxyl­ate (0.15 mol) and tri­phenyl­phosphine (0.15 mol) as coupling reagents in THF (180 ml) at 273 to 298 K. 150 ml of the solvent were evaporated, the residue extracted with toluene/petroleum ether 1/1 and purified by chromatography on silica gel using toluene as eluent to yield 13.6 g (53%) of a colorless oil. The nitrile (0.01 mol) in 30 ml toluene was added to 0.02 mol tri­ethyl­ammonium chloride and 0.02 mol sodium azide and the stirred mixture was heated to reflux for 104 h. Petroleum ether was added and the liquid supernatant was removed. The solid residue was dissolved in warm aqueous ethanol and carefully acidulated with conc. hydro­chloric acid. After cooling on ice, the solid was isolated by suction filtration and recrystallized from ethanol to give colorless crystals with m.p. = 496 K (dec.).

NMR-data of the nitrile: ^1^H-NMR (400 MHz, CDCl_3_) 0.78 (*d*, *J* = 6.8 Hz, 6 H, CH_3_) 0.94–1.13 (*m*, 3 H), 1.43–1.82 (*m*, 5 H), 2.03 (*dq*, *J* = 16 Hz, *J*′= 2.7 Hz, 1 H), 4.68 (*m*, 1 H), 6.90 (*d*, 2 H, 8 Hz) 7.54 (*d*, 2 H, *J* = 8 Hz). ^13^C-NMR (75 MHz, CDCl_3_): 20.7, 21.0, 22.2, 24.8, 26.2, 34.8, 37.4, 47.6, 74.0, 103.1, 115.9 (2 C), 119.4, 134.1 (2 C), 161.7. MS: (EI): 256 (6%, *M*^+^), 82 (100%, C_6_H_10_^+.^).

Analytical data of the title compound: IR (KBr): 2910, 2840, 2222, 1600, 1560, 1482,1450, 1370, 1240, 1075, 1042, 912 cm^−1^; ^1^H-NMR (400 MHz, CDCl_3_ + DMSO-*d*_6_, 20/1): δ = (7.87, *d*, 2 H, ph) (6.90, *d*, 2 H, ph), 5.5–3.0 (*vbs*, 1 N, NH), (4.64, "*s*", 1 H, 1-H neom), 2.00 (*d*, 1H), 1.68 (*m*, 2 H). 1.65–1.44 (*m*, 3 H), 1.1–0.87 (*m*, 3 H), 0.84 (*d*, 3 H, CH_3_), 0.77–0.68 (2*d, isoprop­yl); ^13^C-NMR (100 MHz, CDCl_3_ + DMSO-*d*_6_, 20/1): δ = 160.12, 154.83, 128.52, 115.54, 73.12, 47.02. 387.02, 34.33, 28.80, 25.71, 20.63, 20.33; MS (FD): 792 (2%, *M*_2_^+^), 395.6 (100%, *M*^+^).

## Refinement

Crystal data, data collection and structure refinement details are summarized in Table 2 ▸.

## Supplementary Material

Crystal structure: contains datablock(s) I, global. DOI: 10.1107/S2414314626000337/bt4195sup1.cif

Structure factors: contains datablock(s) I. DOI: 10.1107/S2414314626000337/bt4195Isup2.hkl

CCDC reference: 2522581

Additional supporting information:  crystallographic information; 3D view; checkCIF report

## Figures and Tables

**Table 1:** Hydrogen-bond geometry (Å, °)

*D*—H⋯*A*	*D*—H	H⋯*A*	*D*⋯*A*	*D*—H⋯*A*
N22*A*—H22*A*⋯N19*A*^i^	0.82 (3)	2.08 (3)	2.903 (3)	177 (2)
N22*B*—H22*B*⋯N19*B*^ii^	0.85 (3)	2.05 (3)	2.900 (3)	177 (3)
C14*B*—H14*B*⋯N21*B*^iii^	0.95	2.55	3.493 (3)	170
C14*A*—H14*A*⋯N21*A*^iv^	0.95	2.57	3.495 (3)	165
C16*A*—H16*A*⋯N20*A*^i^	0.95	2.51	3.423 (3)	160
C16*B*—H16*B*⋯N20*B*^ii^	0.95	2.43	3.357 (3)	167

Symmetry codes: (i) 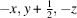; (ii) 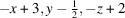; (iii) 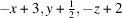; (iv) 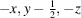.

**Table 2:** Experimental details

Crystal data	
Chemical formula	C_17_H_24_N_4_O
*M*_r_	300.40
Crystal system, space group	Monoclinic, *P*2_1_
Temperature (K)	120
*a*, *b*, *c* (Å)	9.0309 (2), 10.0456 (3), 18.3977 (5)
β (°)	97.431 (2)
*V* (Å^3^)	1655.04 (8)
*Z*	4
Radiation type	Cu *K*α
μ (mm^−1^)	0.61
Crystal size (mm)	0.44 × 0.27 × 0.17

Data collection	
Diffractometer	Stoe Stadivari
No. of measured, independent and observed [*I* > 2σ(*I*)] reflections	12932, 5536, 5198
*R*_int_	0.016
(sin θ/λ)_max_ (Å^−1^)	0.601

Refinement	
*R*[*F*^2^ > 2σ(*F*^2^)], *wR*(*F*^2^), *S*	0.031, 0.082, 1.02
No. of reflections	5536
No. of parameters	411
No. of restraints	1
H-atom treatment	H atoms treated by a mixture of independent and constrained refinement
Δρ_max_, Δρ_min_ (e Å^−3^)	0.15, −0.19
Absolute structure	Flack *x* determined using 2193 quotients [(*I*^+^)−(*I*^−^)]/[(*I*^+^)+(*I*^−^)] (Parsons *et al.*, 2013 ▸)
Absolute structure parameter	0.05 (19)

Computer programs: *X-AREA WinXpose*, *Recipe* and *Integrate*.0 (Stoe & Cie, 2020 ▸), *SHELXT2014* (Sheldrick, 2015*a* ▸), *SHELXL2019/2* (Sheldrick, 2015*b* ▸) and *PLATON* (Spek, 2009 ▸).

## full crystallographic data

### 5-{4-[(1R,3S,4S)-Neomenthyloxy]phenyl}-1H-tetrazole Crystal data

**Table:**

C_17_H_24_N_4_O	*F*(000) = 648
*M**_r_* = 300.40	*D*_x_ = 1.206 Mg m^−^^3^
Monoclinic, *P*2_1_	Cu *K*α radiation, λ = 1.54178 Å
*a* = 9.0309 (2) Å	Cell parameters from 26984 reflections
*b* = 10.0456 (3) Å	θ = 4.9–70.7°
*c* = 18.3977 (5) Å	µ = 0.61 mm^−^^1^
β = 97.431 (2)°	*T* = 120 K
*V* = 1655.04 (8) Å^3^	Block, colorless
*Z* = 4	0.44 × 0.27 × 0.17 mm

### 5-{4-[(1R,3S,4S)-Neomenthyloxy]phenyl}-1H-tetrazole Data collection

**Table:**

Stoe Stadivari diffractometer	5198 reflections with *I* > 2σ(*I*)
Radiation source: microfocus tube	*R*_int_ = 0.016
Detector resolution: 13.33 pixels mm^-1^	θ_max_ = 68.0°, θ_min_ = 4.9°
rotation method, ω scans	*h* = −10→8
12932 measured reflections	*k* = −12→12
5536 independent reflections	*l* = −21→22

### 5-{4-[(1R,3S,4S)-Neomenthyloxy]phenyl}-1H-tetrazole Refinement

**Table:**

Refinement on *F*^2^	Hydrogen site location: mixed
Least-squares matrix: full	H atoms treated by a mixture of independent and constrained refinement
*R*[*F*^2^ > 2σ(*F*^2^)] = 0.031	*w* = 1/[σ^2^(*F*_o_^2^) + (0.0592*P*)^2^] where *P* = (*F*_o_^2^ + 2*F*_c_^2^)/3
*wR*(*F*^2^) = 0.082	(Δ/σ)_max_ < 0.001
*S* = 1.02	Δρ_max_ = 0.15 e Å^−^^3^
5536 reflections	Δρ_min_ = −0.19 e Å^−^^3^
411 parameters	Absolute structure: Flack *x* determined using 2193 quotients [(*I*^+^)-(*I*^-^)]/[(*I*^+^)+(*I*^-^)] (Parsons *et al.*, 2013)
1 restraint	Absolute structure parameter: 0.05 (19)
Primary atom site location: dual	

### 5-{4-[(1R,3S,4S)-Neomenthyloxy]phenyl}-1H-tetrazole Special details

**Table:**

Geometry. All esds (except the esd in the dihedral angle between two l.s. planes) are estimated using the full covariance matrix. The cell esds are taken into account individually in the estimation of esds in distances, angles and torsion angles; correlations between esds in cell parameters are only used when they are defined by crystal symmetry. An approximate (isotropic) treatment of cell esds is used for estimating esds involving l.s. planes.
Refinement. Hydrogen atoms attached to carbons were placed at calculated positions and were refined in the riding-model approximation with C_aromatic_–H = 0.95, C_methyl_–H = 0.98, C_methylene_–H = 0.99, C_tertiary_–H = 1.00 Å, and with *U*_iso_(H) = 1.5 *U*_eq_(C_methyl_) and with *U*_iso_(H) = 1.2 *U*_eq_(C) for the remaining C atoms. The methyl groups were allowed to rotate but not to tip. Hydrogen atoms attached to nitrogen were freely refined with isotropic displacement parameters.

### 5-{4-[(1R,3S,4S)-Neomenthyloxy]phenyl}-1H-tetrazole Fractional atomic coordinates and isotropic or equivalent isotropic displacement parameters (Å^2^)

**Table:**

	*x*	*y*	*z*	*U*_iso_*/*U*_eq_	
C1A	0.5825 (2)	0.5274 (2)	0.29693 (10)	0.0245 (4)	
H1A	0.583779	0.611130	0.267577	0.029*	
C2A	0.4743 (2)	0.5430 (2)	0.35327 (11)	0.0311 (5)	
H2A	0.372802	0.559397	0.327434	0.037*	
H2B	0.503618	0.621710	0.384210	0.037*	
C3A	0.4701 (2)	0.4211 (3)	0.40236 (11)	0.0336 (5)	
H3A	0.430777	0.344572	0.370894	0.040*	
C4A	0.6280 (2)	0.3860 (2)	0.43740 (11)	0.0355 (5)	
H4A	0.664539	0.456434	0.472887	0.043*	
H4B	0.624801	0.301501	0.464859	0.043*	
C5A	0.7369 (2)	0.3713 (2)	0.38170 (11)	0.0302 (5)	
H5A	0.707952	0.293094	0.350269	0.036*	
H5B	0.838055	0.354585	0.407798	0.036*	
C6A	0.7414 (2)	0.49522 (19)	0.33302 (11)	0.0251 (4)	
H6A	0.774833	0.571389	0.366128	0.030*	
C7A	0.3674 (3)	0.4418 (3)	0.46051 (13)	0.0505 (7)	
H7A	0.403677	0.516678	0.492015	0.076*	
H7B	0.366004	0.360955	0.490250	0.076*	
H7C	0.266139	0.461004	0.436801	0.076*	
C8A	0.8528 (2)	0.4834 (2)	0.27747 (12)	0.0322 (5)	
H8A	0.822754	0.405686	0.244945	0.039*	
C9A	0.8538 (3)	0.6064 (3)	0.22948 (13)	0.0477 (6)	
H9A	0.933180	0.598287	0.198140	0.072*	
H9B	0.871610	0.685550	0.260548	0.072*	
H9C	0.757108	0.614834	0.198788	0.072*	
C10A	1.0118 (3)	0.4595 (3)	0.31413 (16)	0.0546 (7)	
H10A	1.014628	0.378564	0.344000	0.082*	
H10B	1.044682	0.535600	0.345468	0.082*	
H10C	1.078438	0.448992	0.276476	0.082*	
O11A	0.53885 (14)	0.41666 (13)	0.24847 (7)	0.0247 (3)	
C12A	0.4188 (2)	0.4282 (2)	0.19580 (10)	0.0209 (4)	
C13A	0.3723 (2)	0.3096 (2)	0.16065 (11)	0.0235 (4)	
H13A	0.424115	0.229221	0.174203	0.028*	
C14A	0.2515 (2)	0.30784 (19)	0.10629 (11)	0.0234 (4)	
H14A	0.220190	0.226123	0.083240	0.028*	
C15A	0.1751 (2)	0.4250 (2)	0.08493 (9)	0.0203 (4)	
C16A	0.2240 (2)	0.54372 (19)	0.11908 (10)	0.0225 (4)	
H16A	0.174227	0.624478	0.104304	0.027*	
C17A	0.3442 (2)	0.5461 (2)	0.17429 (10)	0.0244 (4)	
H17A	0.375490	0.627729	0.197368	0.029*	
C18A	0.0477 (2)	0.42183 (19)	0.02680 (10)	0.0191 (4)	
N19A	−0.01619 (19)	0.31316 (17)	−0.00471 (9)	0.0215 (4)	
N20A	−0.12990 (18)	0.35638 (17)	−0.05509 (8)	0.0232 (4)	
N21A	−0.13654 (18)	0.48508 (17)	−0.05528 (9)	0.0225 (4)	
N22A	−0.02606 (18)	0.52685 (19)	−0.00412 (9)	0.0199 (4)	
H22A	−0.014 (3)	0.608 (3)	−0.0001 (13)	0.021 (6)*	
C1B	0.9115 (2)	0.4506 (2)	0.71274 (10)	0.0238 (4)	
H1B	0.995759	0.385528	0.715363	0.029*	
C2B	0.7796 (2)	0.38651 (19)	0.74236 (10)	0.0258 (4)	
H2C	0.809383	0.359731	0.793965	0.031*	
H2D	0.749666	0.305300	0.713677	0.031*	
C3B	0.6469 (2)	0.4817 (2)	0.73827 (11)	0.0285 (4)	
H3B	0.679123	0.561408	0.768874	0.034*	
C4B	0.6058 (2)	0.5288 (2)	0.65946 (11)	0.0304 (5)	
H4C	0.523248	0.593969	0.657567	0.036*	
H4D	0.569917	0.451856	0.628417	0.036*	
C5B	0.7376 (2)	0.5928 (2)	0.62863 (11)	0.0291 (5)	
H5C	0.765557	0.675987	0.655890	0.035*	
H5D	0.707064	0.616626	0.576635	0.035*	
C6B	0.8740 (2)	0.5006 (2)	0.63389 (10)	0.0261 (4)	
H6B	0.845263	0.421292	0.602306	0.031*	
C7B	0.5149 (3)	0.4194 (3)	0.76912 (14)	0.0425 (6)	
H7D	0.546085	0.390597	0.819692	0.064*	
H7E	0.479181	0.342292	0.739219	0.064*	
H7F	0.434461	0.484983	0.768353	0.064*	
C8B	1.0097 (2)	0.5657 (3)	0.60540 (11)	0.0340 (5)	
H8B	1.034735	0.647748	0.635275	0.041*	
C9B	1.1473 (3)	0.4767 (3)	0.61509 (15)	0.0548 (7)	
H9D	1.124336	0.392246	0.589452	0.082*	
H9E	1.176890	0.459495	0.667379	0.082*	
H9F	1.229293	0.521156	0.594664	0.082*	
C10B	0.9761 (3)	0.6092 (3)	0.52553 (13)	0.0492 (7)	
H10D	1.064929	0.651043	0.510099	0.074*	
H10E	0.893426	0.673132	0.520477	0.074*	
H10F	0.948229	0.531286	0.494696	0.074*	
O11B	0.95890 (14)	0.56759 (14)	0.75611 (7)	0.0245 (3)	
C12B	1.0721 (2)	0.5587 (2)	0.81188 (9)	0.0201 (4)	
C13B	1.1269 (2)	0.6803 (2)	0.84092 (11)	0.0226 (4)	
H13B	1.080327	0.760915	0.823272	0.027*	
C14B	1.2479 (2)	0.68448 (19)	0.89494 (11)	0.0225 (4)	
H14B	1.285838	0.767792	0.913345	0.027*	
C15B	1.3148 (2)	0.5663 (2)	0.92267 (9)	0.0189 (4)	
C16B	1.2553 (2)	0.44547 (19)	0.89608 (10)	0.0201 (4)	
H16B	1.298066	0.364810	0.915915	0.024*	
C17B	1.1349 (2)	0.4403 (2)	0.84129 (10)	0.0214 (4)	
H17B	1.095384	0.356950	0.823855	0.026*	
C18B	1.4446 (2)	0.57123 (19)	0.97906 (10)	0.0190 (4)	
N19B	1.5107 (2)	0.67937 (17)	1.00894 (9)	0.0225 (4)	
N20B	1.62635 (18)	0.63602 (17)	1.05854 (9)	0.0245 (4)	
N21B	1.63188 (18)	0.50794 (16)	1.05952 (9)	0.0234 (4)	
N22B	1.51881 (18)	0.46519 (18)	1.00996 (9)	0.0200 (4)	
H22B	1.507 (3)	0.381 (3)	1.0051 (14)	0.040 (7)*	

### 5-{4-[(1R,3S,4S)-Neomenthyloxy]phenyl}-1H-tetrazole Atomic displacement parameters (Å^2^)

**Table:**

	*U*^11^	*U*^22^	*U*^33^	*U*^12^	*U*^13^	*U*^23^
C1A	0.0255 (11)	0.0209 (10)	0.0253 (9)	0.0005 (7)	−0.0040 (8)	−0.0030 (8)
C2A	0.0252 (10)	0.0350 (11)	0.0308 (10)	0.0058 (8)	−0.0046 (8)	−0.0088 (9)
C3A	0.0248 (11)	0.0467 (13)	0.0290 (10)	−0.0043 (9)	0.0020 (8)	−0.0024 (10)
C4A	0.0308 (12)	0.0433 (12)	0.0308 (10)	−0.0023 (9)	−0.0021 (9)	0.0090 (9)
C5A	0.0236 (11)	0.0300 (11)	0.0350 (11)	0.0018 (8)	−0.0040 (8)	0.0067 (9)
C6A	0.0233 (10)	0.0210 (10)	0.0294 (10)	−0.0002 (7)	−0.0028 (8)	−0.0010 (8)
C7A	0.0356 (14)	0.082 (2)	0.0345 (12)	−0.0028 (13)	0.0052 (10)	−0.0052 (13)
C8A	0.0268 (11)	0.0306 (11)	0.0391 (12)	−0.0052 (8)	0.0033 (9)	−0.0045 (9)
C9A	0.0418 (15)	0.0646 (18)	0.0365 (12)	−0.0042 (11)	0.0040 (10)	0.0151 (12)
C10A	0.0286 (13)	0.0625 (18)	0.0742 (18)	0.0049 (11)	0.0125 (13)	0.0311 (15)
O11A	0.0261 (8)	0.0205 (7)	0.0255 (7)	0.0029 (6)	−0.0041 (5)	−0.0016 (6)
C12A	0.0198 (10)	0.0229 (10)	0.0197 (9)	−0.0006 (8)	0.0011 (7)	0.0003 (8)
C13A	0.0230 (11)	0.0182 (10)	0.0290 (10)	0.0018 (8)	0.0023 (8)	0.0038 (8)
C14A	0.0241 (10)	0.0177 (10)	0.0277 (10)	−0.0026 (8)	0.0009 (8)	−0.0019 (8)
C15A	0.0207 (10)	0.0199 (9)	0.0208 (9)	−0.0012 (8)	0.0044 (7)	0.0012 (8)
C16A	0.0261 (10)	0.0163 (9)	0.0244 (9)	0.0029 (7)	0.0008 (7)	0.0004 (7)
C17A	0.0273 (11)	0.0194 (10)	0.0251 (9)	−0.0003 (8)	−0.0014 (8)	−0.0048 (8)
C18A	0.0207 (10)	0.0165 (9)	0.0205 (9)	−0.0008 (7)	0.0046 (7)	0.0010 (8)
N19A	0.0219 (9)	0.0179 (8)	0.0239 (8)	−0.0014 (6)	−0.0003 (6)	0.0020 (6)
N20A	0.0242 (9)	0.0200 (9)	0.0244 (8)	−0.0017 (6)	−0.0004 (7)	0.0023 (7)
N21A	0.0221 (9)	0.0207 (9)	0.0242 (8)	−0.0022 (6)	0.0005 (7)	−0.0005 (6)
N22A	0.0206 (9)	0.0156 (9)	0.0230 (8)	−0.0011 (6)	0.0008 (6)	−0.0004 (6)
C1B	0.0218 (10)	0.0247 (11)	0.0240 (9)	0.0020 (8)	−0.0009 (7)	−0.0051 (8)
C2B	0.0316 (11)	0.0216 (9)	0.0233 (9)	−0.0008 (8)	0.0008 (8)	−0.0010 (7)
C3B	0.0252 (11)	0.0276 (10)	0.0337 (10)	−0.0010 (8)	0.0080 (8)	−0.0038 (8)
C4B	0.0195 (10)	0.0306 (10)	0.0401 (11)	0.0016 (7)	0.0000 (8)	0.0019 (9)
C5B	0.0254 (11)	0.0328 (12)	0.0277 (10)	−0.0017 (8)	−0.0024 (8)	0.0050 (8)
C6B	0.0206 (10)	0.0343 (11)	0.0227 (9)	−0.0038 (8)	0.0002 (7)	−0.0039 (8)
C7B	0.0365 (13)	0.0424 (13)	0.0519 (13)	−0.0044 (10)	0.0181 (11)	0.0010 (11)
C8B	0.0278 (12)	0.0478 (13)	0.0264 (10)	−0.0075 (10)	0.0037 (8)	−0.0071 (10)
C9B	0.0291 (14)	0.090 (2)	0.0480 (14)	0.0017 (13)	0.0137 (11)	−0.0001 (14)
C10B	0.0425 (15)	0.0715 (18)	0.0345 (12)	−0.0191 (12)	0.0087 (10)	0.0042 (12)
O11B	0.0235 (7)	0.0237 (7)	0.0242 (6)	0.0017 (6)	−0.0050 (5)	−0.0034 (6)
C12B	0.0171 (9)	0.0238 (10)	0.0199 (8)	−0.0001 (8)	0.0036 (7)	0.0009 (8)
C13B	0.0232 (11)	0.0174 (10)	0.0268 (10)	0.0012 (7)	0.0017 (8)	0.0037 (8)
C14B	0.0230 (10)	0.0167 (9)	0.0273 (10)	−0.0025 (7)	0.0016 (8)	0.0007 (8)
C15B	0.0178 (9)	0.0187 (9)	0.0206 (8)	−0.0017 (8)	0.0033 (7)	−0.0002 (8)
C16B	0.0209 (10)	0.0175 (9)	0.0223 (9)	0.0022 (7)	0.0038 (7)	0.0013 (7)
C17B	0.0222 (10)	0.0178 (9)	0.0242 (9)	−0.0028 (7)	0.0034 (7)	−0.0038 (7)
C18B	0.0191 (9)	0.0157 (9)	0.0227 (8)	0.0001 (8)	0.0044 (7)	0.0016 (8)
N19B	0.0215 (8)	0.0179 (8)	0.0266 (9)	−0.0015 (6)	−0.0025 (7)	0.0003 (6)
N20B	0.0237 (9)	0.0187 (9)	0.0292 (9)	−0.0006 (6)	−0.0038 (7)	0.0016 (7)
N21B	0.0226 (9)	0.0169 (9)	0.0289 (9)	−0.0012 (6)	−0.0038 (7)	0.0003 (6)
N22B	0.0193 (9)	0.0135 (9)	0.0260 (8)	−0.0017 (6)	−0.0011 (7)	−0.0007 (6)

### 5-{4-[(1R,3S,4S)-Neomenthyloxy]phenyl}-1H-tetrazole Geometric parameters (Å, º)

**Table:**

C1A—O11A	1.448 (2)	C1B—O11B	1.454 (2)
C1A—C2A	1.522 (3)	C1B—C2B	1.516 (3)
C1A—C6A	1.536 (3)	C1B—C6B	1.531 (3)
C1A—H1A	1.0000	C1B—H1B	1.0000
C2A—C3A	1.525 (3)	C2B—C3B	1.527 (3)
C2A—H2A	0.9900	C2B—H2C	0.9900
C2A—H2B	0.9900	C2B—H2D	0.9900
C3A—C7A	1.518 (3)	C3B—C7B	1.520 (3)
C3A—C4A	1.528 (3)	C3B—C4B	1.525 (3)
C3A—H3A	1.0000	C3B—H3B	1.0000
C4A—C5A	1.516 (3)	C4B—C5B	1.525 (3)
C4A—H4A	0.9900	C4B—H4C	0.9900
C4A—H4B	0.9900	C4B—H4D	0.9900
C5A—C6A	1.538 (3)	C5B—C6B	1.535 (3)
C5A—H5A	0.9900	C5B—H5C	0.9900
C5A—H5B	0.9900	C5B—H5D	0.9900
C6A—C8A	1.529 (3)	C6B—C8B	1.540 (3)
C6A—H6A	1.0000	C6B—H6B	1.0000
C7A—H7A	0.9800	C7B—H7D	0.9800
C7A—H7B	0.9800	C7B—H7E	0.9800
C7A—H7C	0.9800	C7B—H7F	0.9800
C8A—C9A	1.519 (3)	C8B—C9B	1.523 (4)
C8A—C10A	1.524 (3)	C8B—C10B	1.525 (3)
C8A—H8A	1.0000	C8B—H8B	1.0000
C9A—H9A	0.9800	C9B—H9D	0.9800
C9A—H9B	0.9800	C9B—H9E	0.9800
C9A—H9C	0.9800	C9B—H9F	0.9800
C10A—H10A	0.9800	C10B—H10D	0.9800
C10A—H10B	0.9800	C10B—H10E	0.9800
C10A—H10C	0.9800	C10B—H10F	0.9800
O11A—C12A	1.362 (2)	O11B—C12B	1.355 (2)
C12A—C13A	1.394 (3)	C12B—C17B	1.396 (3)
C12A—C17A	1.395 (3)	C12B—C13B	1.398 (3)
C13A—C14A	1.381 (3)	C13B—C14B	1.379 (3)
C13A—H13A	0.9500	C13B—H13B	0.9500
C14A—C15A	1.395 (3)	C14B—C15B	1.398 (3)
C14A—H14A	0.9500	C14B—H14B	0.9500
C15A—C16A	1.393 (3)	C15B—C16B	1.390 (3)
C15A—C18A	1.466 (3)	C15B—C18B	1.462 (2)
C16A—C17A	1.387 (3)	C16B—C17B	1.384 (3)
C16A—H16A	0.9500	C16B—H16B	0.9500
C17A—H17A	0.9500	C17B—H17B	0.9500
C18A—N19A	1.332 (3)	C18B—N19B	1.324 (3)
C18A—N22A	1.335 (3)	C18B—N22B	1.345 (3)
N19A—N20A	1.362 (2)	N19B—N20B	1.366 (2)
N20A—N21A	1.294 (2)	N20B—N21B	1.288 (2)
N21A—N22A	1.347 (2)	N21B—N22B	1.348 (2)
N22A—H22A	0.82 (3)	N22B—H22B	0.85 (3)
			
O11A—C1A—C2A	110.53 (16)	O11B—C1B—C2B	109.61 (15)
O11A—C1A—C6A	105.59 (15)	O11B—C1B—C6B	105.58 (15)
C2A—C1A—C6A	111.94 (16)	C2B—C1B—C6B	113.09 (16)
O11A—C1A—H1A	109.6	O11B—C1B—H1B	109.5
C2A—C1A—H1A	109.6	C2B—C1B—H1B	109.5
C6A—C1A—H1A	109.6	C6B—C1B—H1B	109.5
C1A—C2A—C3A	112.83 (17)	C1B—C2B—C3B	111.35 (16)
C1A—C2A—H2A	109.0	C1B—C2B—H2C	109.4
C3A—C2A—H2A	109.0	C3B—C2B—H2C	109.4
C1A—C2A—H2B	109.0	C1B—C2B—H2D	109.4
C3A—C2A—H2B	109.0	C3B—C2B—H2D	109.4
H2A—C2A—H2B	107.8	H2C—C2B—H2D	108.0
C7A—C3A—C2A	111.8 (2)	C7B—C3B—C4B	112.13 (18)
C7A—C3A—C4A	110.87 (17)	C7B—C3B—C2B	111.82 (18)
C2A—C3A—C4A	110.03 (18)	C4B—C3B—C2B	109.50 (16)
C7A—C3A—H3A	108.0	C7B—C3B—H3B	107.7
C2A—C3A—H3A	108.0	C4B—C3B—H3B	107.7
C4A—C3A—H3A	108.0	C2B—C3B—H3B	107.7
C5A—C4A—C3A	112.86 (16)	C3B—C4B—C5B	112.26 (16)
C5A—C4A—H4A	109.0	C3B—C4B—H4C	109.2
C3A—C4A—H4A	109.0	C5B—C4B—H4C	109.2
C5A—C4A—H4B	109.0	C3B—C4B—H4D	109.2
C3A—C4A—H4B	109.0	C5B—C4B—H4D	109.2
H4A—C4A—H4B	107.8	H4C—C4B—H4D	107.9
C4A—C5A—C6A	112.53 (18)	C4B—C5B—C6B	112.33 (16)
C4A—C5A—H5A	109.1	C4B—C5B—H5C	109.1
C6A—C5A—H5A	109.1	C6B—C5B—H5C	109.1
C4A—C5A—H5B	109.1	C4B—C5B—H5D	109.1
C6A—C5A—H5B	109.1	C6B—C5B—H5D	109.1
H5A—C5A—H5B	107.8	H5C—C5B—H5D	107.9
C8A—C6A—C1A	112.73 (16)	C1B—C6B—C5B	109.57 (16)
C8A—C6A—C5A	113.34 (17)	C1B—C6B—C8B	111.95 (16)
C1A—C6A—C5A	109.19 (16)	C5B—C6B—C8B	112.91 (18)
C8A—C6A—H6A	107.1	C1B—C6B—H6B	107.4
C1A—C6A—H6A	107.1	C5B—C6B—H6B	107.4
C5A—C6A—H6A	107.1	C8B—C6B—H6B	107.4
C3A—C7A—H7A	109.5	C3B—C7B—H7D	109.5
C3A—C7A—H7B	109.5	C3B—C7B—H7E	109.5
H7A—C7A—H7B	109.5	H7D—C7B—H7E	109.5
C3A—C7A—H7C	109.5	C3B—C7B—H7F	109.5
H7A—C7A—H7C	109.5	H7D—C7B—H7F	109.5
H7B—C7A—H7C	109.5	H7E—C7B—H7F	109.5
C9A—C8A—C10A	108.04 (19)	C9B—C8B—C10B	109.8 (2)
C9A—C8A—C6A	112.28 (19)	C9B—C8B—C6B	112.6 (2)
C10A—C8A—C6A	112.35 (19)	C10B—C8B—C6B	112.42 (17)
C9A—C8A—H8A	108.0	C9B—C8B—H8B	107.2
C10A—C8A—H8A	108.0	C10B—C8B—H8B	107.2
C6A—C8A—H8A	108.0	C6B—C8B—H8B	107.2
C8A—C9A—H9A	109.5	C8B—C9B—H9D	109.5
C8A—C9A—H9B	109.5	C8B—C9B—H9E	109.5
H9A—C9A—H9B	109.5	H9D—C9B—H9E	109.5
C8A—C9A—H9C	109.5	C8B—C9B—H9F	109.5
H9A—C9A—H9C	109.5	H9D—C9B—H9F	109.5
H9B—C9A—H9C	109.5	H9E—C9B—H9F	109.5
C8A—C10A—H10A	109.5	C8B—C10B—H10D	109.5
C8A—C10A—H10B	109.5	C8B—C10B—H10E	109.5
H10A—C10A—H10B	109.5	H10D—C10B—H10E	109.5
C8A—C10A—H10C	109.5	C8B—C10B—H10F	109.5
H10A—C10A—H10C	109.5	H10D—C10B—H10F	109.5
H10B—C10A—H10C	109.5	H10E—C10B—H10F	109.5
C12A—O11A—C1A	120.05 (15)	C12B—O11B—C1B	120.18 (15)
O11A—C12A—C13A	114.95 (17)	O11B—C12B—C17B	125.35 (18)
O11A—C12A—C17A	125.80 (17)	O11B—C12B—C13B	115.29 (17)
C13A—C12A—C17A	119.23 (16)	C17B—C12B—C13B	119.36 (16)
C14A—C13A—C12A	120.60 (18)	C14B—C13B—C12B	120.67 (18)
C14A—C13A—H13A	119.7	C14B—C13B—H13B	119.7
C12A—C13A—H13A	119.7	C12B—C13B—H13B	119.7
C13A—C14A—C15A	120.59 (18)	C13B—C14B—C15B	120.12 (18)
C13A—C14A—H14A	119.7	C13B—C14B—H14B	119.9
C15A—C14A—H14A	119.7	C15B—C14B—H14B	119.9
C16A—C15A—C14A	118.61 (16)	C16B—C15B—C14B	118.93 (16)
C16A—C15A—C18A	121.38 (17)	C16B—C15B—C18B	121.14 (18)
C14A—C15A—C18A	120.00 (17)	C14B—C15B—C18B	119.93 (18)
C17A—C16A—C15A	121.14 (17)	C17B—C16B—C15B	121.33 (17)
C17A—C16A—H16A	119.4	C17B—C16B—H16B	119.3
C15A—C16A—H16A	119.4	C15B—C16B—H16B	119.3
C16A—C17A—C12A	119.81 (17)	C16B—C17B—C12B	119.46 (18)
C16A—C17A—H17A	120.1	C16B—C17B—H17B	120.3
C12A—C17A—H17A	120.1	C12B—C17B—H17B	120.3
N19A—C18A—N22A	107.33 (16)	N19B—C18B—N22B	107.53 (16)
N19A—C18A—C15A	126.16 (17)	N19B—C18B—C15B	126.82 (18)
N22A—C18A—C15A	126.50 (17)	N22B—C18B—C15B	125.66 (18)
C18A—N19A—N20A	106.31 (16)	C18B—N19B—N20B	106.28 (16)
N21A—N20A—N19A	110.54 (15)	N21B—N20B—N19B	110.65 (16)
N20A—N21A—N22A	106.22 (16)	N20B—N21B—N22B	106.51 (16)
C18A—N22A—N21A	109.59 (17)	C18B—N22B—N21B	109.03 (17)
C18A—N22A—H22A	133.5 (16)	C18B—N22B—H22B	133.7 (18)
N21A—N22A—H22A	116.7 (16)	N21B—N22B—H22B	117.3 (18)
			
O11A—C1A—C2A—C3A	61.3 (2)	O11B—C1B—C2B—C3B	60.82 (19)
C6A—C1A—C2A—C3A	−56.1 (2)	C6B—C1B—C2B—C3B	−56.7 (2)
C1A—C2A—C3A—C7A	177.13 (17)	C1B—C2B—C3B—C7B	−178.96 (17)
C1A—C2A—C3A—C4A	53.5 (2)	C1B—C2B—C3B—C4B	56.1 (2)
C7A—C3A—C4A—C5A	−177.1 (2)	C7B—C3B—C4B—C5B	179.34 (18)
C2A—C3A—C4A—C5A	−52.9 (3)	C2B—C3B—C4B—C5B	−55.9 (2)
C3A—C4A—C5A—C6A	55.0 (2)	C3B—C4B—C5B—C6B	55.4 (2)
O11A—C1A—C6A—C8A	61.4 (2)	O11B—C1B—C6B—C5B	−66.18 (19)
C2A—C1A—C6A—C8A	−178.22 (17)	C2B—C1B—C6B—C5B	53.7 (2)
O11A—C1A—C6A—C5A	−65.5 (2)	O11B—C1B—C6B—C8B	59.9 (2)
C2A—C1A—C6A—C5A	54.9 (2)	C2B—C1B—C6B—C8B	179.73 (17)
C4A—C5A—C6A—C8A	178.85 (16)	C4B—C5B—C6B—C1B	−52.5 (2)
C4A—C5A—C6A—C1A	−54.6 (2)	C4B—C5B—C6B—C8B	−178.03 (16)
C1A—C6A—C8A—C9A	54.6 (2)	C1B—C6B—C8B—C9B	52.3 (2)
C5A—C6A—C8A—C9A	179.30 (17)	C5B—C6B—C8B—C9B	176.50 (18)
C1A—C6A—C8A—C10A	176.65 (19)	C1B—C6B—C8B—C10B	176.9 (2)
C5A—C6A—C8A—C10A	−58.7 (2)	C5B—C6B—C8B—C10B	−58.9 (3)
C2A—C1A—O11A—C12A	74.1 (2)	C2B—C1B—O11B—C12B	95.80 (18)
C6A—C1A—O11A—C12A	−164.63 (15)	C6B—C1B—O11B—C12B	−142.09 (16)
C1A—O11A—C12A—C13A	−169.86 (16)	C1B—O11B—C12B—C17B	−11.6 (3)
C1A—O11A—C12A—C17A	11.6 (3)	C1B—O11B—C12B—C13B	168.60 (16)
O11A—C12A—C13A—C14A	179.84 (17)	O11B—C12B—C13B—C14B	−176.15 (17)
C17A—C12A—C13A—C14A	−1.5 (3)	C17B—C12B—C13B—C14B	4.1 (3)
C12A—C13A—C14A—C15A	0.8 (3)	C12B—C13B—C14B—C15B	−1.7 (3)
C13A—C14A—C15A—C16A	0.6 (3)	C13B—C14B—C15B—C16B	−1.4 (3)
C13A—C14A—C15A—C18A	179.56 (17)	C13B—C14B—C15B—C18B	178.97 (17)
C14A—C15A—C16A—C17A	−1.3 (3)	C14B—C15B—C16B—C17B	2.2 (3)
C18A—C15A—C16A—C17A	179.71 (18)	C18B—C15B—C16B—C17B	−178.23 (17)
C15A—C16A—C17A—C12A	0.7 (3)	C15B—C16B—C17B—C12B	0.2 (3)
O11A—C12A—C17A—C16A	179.27 (17)	O11B—C12B—C17B—C16B	176.94 (17)
C13A—C12A—C17A—C16A	0.8 (3)	C13B—C12B—C17B—C16B	−3.3 (3)
C16A—C15A—C18A—N19A	−173.60 (18)	C16B—C15B—C18B—N19B	−179.59 (18)
C14A—C15A—C18A—N19A	7.5 (3)	C14B—C15B—C18B—N19B	0.0 (3)
C16A—C15A—C18A—N22A	6.1 (3)	C16B—C15B—C18B—N22B	0.5 (3)
C14A—C15A—C18A—N22A	−172.88 (18)	C14B—C15B—C18B—N22B	−179.89 (17)
N22A—C18A—N19A—N20A	0.0 (2)	N22B—C18B—N19B—N20B	0.0 (2)
C15A—C18A—N19A—N20A	179.67 (17)	C15B—C18B—N19B—N20B	−179.91 (18)
C18A—N19A—N20A—N21A	0.2 (2)	C18B—N19B—N20B—N21B	0.0 (2)
N19A—N20A—N21A—N22A	−0.2 (2)	N19B—N20B—N21B—N22B	0.1 (2)
N19A—C18A—N22A—N21A	−0.1 (2)	N19B—C18B—N22B—N21B	0.1 (2)
C15A—C18A—N22A—N21A	−179.82 (17)	C15B—C18B—N22B—N21B	179.97 (17)
N20A—N21A—N22A—C18A	0.2 (2)	N20B—N21B—N22B—C18B	−0.1 (2)

### 5-{4-[(1R,3S,4S)-Neomenthyloxy]phenyl}-1H-tetrazole Hydrogen-bond geometry (Å, º)

**Table:**

*D*—H···*A*	*D*—H	H···*A*	*D*···*A*	*D*—H···*A*
N22*A*—H22*A*···N19*A*^i^	0.82 (3)	2.08 (3)	2.903 (3)	177 (2)
N22*B*—H22*B*···N19*B*^ii^	0.85 (3)	2.05 (3)	2.900 (3)	177 (3)
C14*B*—H14*B*···N21*B*^iii^	0.95	2.55	3.493 (3)	170
C14*A*—H14*A*···N21*A*^iv^	0.95	2.57	3.495 (3)	165
C16*A*—H16*A*···N20*A*^i^	0.95	2.51	3.423 (3)	160
C16*B*—H16*B*···N20*B*^ii^	0.95	2.43	3.357 (3)	167

Symmetry codes: (i) −*x*, *y*+1/2, −*z*; (ii) −*x*+3, *y*−1/2, −*z*+2; (iii) −*x*+3, *y*+1/2, −*z*+2; (iv) −*x*, *y*−1/2, −*z*.
